# High phosphorus intake and gut-related parameters – results of a randomized placebo-controlled human intervention study

**DOI:** 10.1186/s12937-018-0331-4

**Published:** 2018-02-16

**Authors:** Ulrike Trautvetter, Amélia Camarinha-Silva, Gerhard Jahreis, Stefan Lorkowski, Michael Glei

**Affiliations:** 1 Department of Nutritional Toxicology, Institute of Nutrition, Friedrich Schiller University Jena, Dornburger Straße 24, 07743 Jena, Germany; 2 Institute of Animal Science, University of Hohenheim, Emil-Wolff-Straße. 10, 70599 Stuttgart, Germany; 3 Department of Nutritional Physiology, Institute of Nutrition, Friedrich Schiller University Jena, Dornburger Straße 23, 07743 Jena, Germany; 4 Department of Nutritional Biochemistry and Physiology, Institute of Nutrition, Friedrich Schiller University Jena, Dornburger Straße 25, 07743 Jena, Germany; 5 Competence Cluster for Nutrition and Cardiovascular Health (nutriCARD) Halle-Jena-Leipzig, Jena, Germany

**Keywords:** Phosphorus intake, Phosphate intake, Calcium intake, Human study, Faecal water, Cytotoxicity, Genotoxicity, Short-chain fatty acids

## Abstract

**Background:**

In recent years, high phosphate intakes were discussed critically. In the small intestine, a part of the ingested phosphate and calcium precipitates to amorphous calcium phosphate (ACP), which in turn can precipitate other intestinal substances, thus leading to a beneficial modulation of the intestinal environment. Therefore, we analysed faecal samples obtained from a human intervention study regarding gut-related parameters.

**Methods:**

Sixty-two healthy subjects (men, *n* = 30; women, *n* = 32) completed the double-blind, placebo-controlled and parallel designed study (mean age: 29 ± 7 years; mean BMI: 24 ± 3 kg/m^2^). Supplements were monosodium phosphate and calcium carbonate. During the first 2 weeks, all groups consumed a placebo sherbet powder, and afterwards a sherbet powder for 8 weeks according to the intervention group: P1000/Ca0 (1000 mg/d phosphorus), P1000/Ca500 (1000 mg/d phosphorus and 500 mg/d calcium) and P1000/Ca1000 (1000 mg/d phosphorus and 1000 mg/d calcium). After the placebo period and after 8 weeks of intervention faecal collections took place. We determined in faeces: short-chain fatty acids (SCFA) and fat as well as the composition of the microbiome (subgroup) and cyto- and genotoxicity of faecal water (FW). By questionnaire evaluation we examined tolerability of the used phosphorus supplement.

**Results:**

Faecal fat concentrations did not change significantly due to the interventions. Concentrations of faecal total SCFA and acetate were significantly higher after 8 weeks of P1000/Ca500 supplementation compared to the P1000/Ca0 supplementation. In men, faecal total SCFA and acetate concentrations were significantly higher after 8 weeks in the P1000/Ca1000 group compared to the P1000/Ca0 one. None of the interventions markedly affected cyto- and genotoxic activity of FW. Men of the P1000/Ca1000 intervention had a significantly different gut microbial community compared to the men of the P1000/Ca0 and P1000/Ca500 ones. The genus *Clostridium* XVIII was significantly more abundant in men of the P1000/Ca1000 intervention group compared to the other groups. Supplementations did not cause increased intestinal distress.

**Conclusions:**

The used high phosphorus diet did not influence cyto- and genotoxicity of FW and the concentrations of faecal fat independent of calcium intake. Our study provides first hints for a potential phosphorus-induced modulation of the gut community and the faecal total SCFA content.

**Trial registration:**

The trial is registered at ClinicalTrials.gov as NCT02095392.

**Electronic supplementary material:**

The online version of this article (10.1186/s12937-018-0331-4) contains supplementary material, which is available to authorized users.

## Background

Dietary phosphate and serum phosphate concentrations of healthy people and patients with chronic kidney disease have been discussed critically in the last years, regarding bone, cardiovascular health and mortality [1–4]. Whereas associations of phosphate concentrations in serum with cardiovascular diseases and mortality have been well established [2], it is still unclear whether dietary phosphorus influences serum phosphate concentrations [5, 6].

According to the National Health and Nutrition Examination Survey of the USA, dietary phosphorus intake of Americans exceeds the daily recommended intake of 700 mg phosphorus for adults and the elderly [4, 7]. This is mainly a result of the increased consumption of food prepared or treated with phosphate additives, which ranged from baked goods to cola beverages [8, 9]. In 2014, our department determined the influence of a high phosphorus intake in combination with different calcium supplies on phosphorus, calcium, magnesium and iron metabolism as well as fibroblast growth factor 23 in a human intervention study [10]. In this and in other intervention studies, increased concentrations of phosphorus and calcium in the faeces after high calcium and phosphorus supplementation compared to placebo were found [10–13]. However, up to now, only a few studies dealt with the question, which effects are provoked by higher intestinal concentrations of calcium and phosphorus in the human gut. It is known that calcium and phosphate form amorphous calcium phosphate complexes (ACP) in the small intestine. Intestinal substances and food ingredients, such as bile and fatty acids, can bind to ACP [13, 14]. This leads to several physiological changes in the human gut, e.g. a modulation of the composition of faecal bile acids, faecal short-chain fatty acids (SCFA) and gut microbiota [11, 13]. Furthermore, high phosphorus intakes can lead to disturbances in the gut, like diarrhoea [15].

In the study presented here, we analysed faecal samples from the subjects of the above-mentioned study [10] regarding cytotoxicity and genotoxicity of faecal water (FW), faecal concentrations of SCFA and fat as well as the composition of the gut microbiome (in a subgroup of study subjects). In addition, we examined the tolerability of the used phosphorus supplement with respect to gut distress, such as diarrhoea or stomach ache, by questionnaire evaluation.

## Methods

### Supplements

In the current study, two supplements were used: monosodium phosphate (NaH_2_PO_4_; cfb, Budenheim, Germany) and calcium carbonate (CaCO_3;_ cfb, Budenheim, Germany). In order to achieve a supplementation of additional 1000 mg phosphorus/d as well as additional 500 or 1000 mg calcium/d, monosodium phosphate and calcium carbonate were mixed with sherbet powder. Sherbet powder without additional supplements served as placebo. Participants were encouraged to drink the sherbet powder twice a day diluted in 250 ml water and to document when the sherbet powder was not consumed. Detailed information has been published elsewhere [10].

### Subjects and study design

The study was conducted by the Institute of Nutrition of the Friedrich Schiller University Jena between March and July 2014.

Sixty-six omnivorous healthy subjects (men, *n* = 33; women *n* = 33) participated in this double-blinded, placebo-controlled and parallel designed study. Eligibility criteria for participants were an age between 18 and 60 years as well as mental and physical well-being. Exclusion criteria were regular intake of dietary supplements, renal diseases, pregnancy, nursing as well as post-menopausal age. Renal diseases were determined with the Chronic Kidney Disease Epidemiology Collaboration equation for estimating the glomerular filtration rate [16]. Subjects with baseline glomerular filtration rates of < 80 ml/min/1.73 m^2^ were excluded.

The volunteers were provided with detailed information regarding purpose, course, and possible risks of the study. This study was conducted according to the guidelines laid down in the Declaration of Helsinki and all procedures involving human subjects were approved by the Ethical Committee of the Friedrich Schiller University Jena (no. 3987-01/14). Written informed consent was obtained from all subjects. The trial is registered at ClinicalTrials.gov as NCT02095392. Four participants dropped out because of illness and personal reasons. The remaining 62 volunteers (men, *n* = 30; women, *n* = 32) aged 29 ± 7 years and had a mean BMI of 24 ± 3 kg/m^2^.

**Fig. 1:**
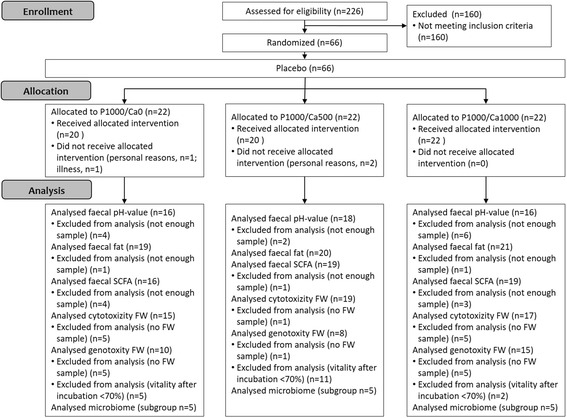
Flow chart of the study concept. P1000/Ca0: 1000 mg phosphorus; P1000/Ca500: 1000 mg phosphorus/500 mg calcium; P1000/Ca1000: 1000 mg phosphorus/1000 mg calcium; SCFA: short-chain fatty acids; FW: faecal water

The details of the study design have been published previously [10]. In short, 62 healthy subjects completed a double-blinded, placebo-controlled and parallel designed study. During the first two weeks, all groups consumed a placebo sherbet powder and afterwards, for eight weeks, a supplemented sherbet powder according to the intervention group: P1000/Ca0 (1000 mg/d phosphorus), P1000/Ca500 (1000 mg/d phosphorus and 500 mg/d calcium) and P1000/Ca1000 (1000 mg/d phosphorus and 1000 mg/d calcium). Dietary records, fasting blood samplings and urine collections took place (after placebo, four and eight weeks of supplementation). After two weeks of placebo and eight weeks of intervention subjects collected one faeces sample. Figure 1 shows the flow chart with the number of subjects included and excluded on each analysis.

### Supplement tolerance questionnaire

After placebo as well as after four and eight weeks of supplementation, subjects were encouraged to fill out a questionnaire about diverse health aspects for the last weeks. One question aimed to assess complaints regarding gut health. The subjects should report, whether they had problems with diarrhoea, obstipation, flatulence or undefined stomach ache. In the case of such problems, the subjects were asked to state, whether these problems could be due to the consumption of the test products (supplemented or non-supplemented powder).

### Faecal analysis

At the evening before or at the morning of the blood sampling, the subjects were encouraged to collect one whole defecation in provided boxes and to store the sample in a cool dark place. The faecal samples were transported to the study centre at the day of the blood sampling after placebo and eight weeks of intervention. Each specimen was weighed and homogenised. Faecal pH value was measured using a glass pH electrode (InLab 420 electrode MP 225, Mettler Toledo GmbH, Giessen, Germany). Faeces samples were aliquoted for the respective analysis (faecal fat and FW preparation) and stored at − 20 °C until use. For SCFA analysis, 1 g fresh faeces were diluted with 2 ml distilled water, mixed and stored at − 20 °C until use.

### Faecal fat

Faecal fat was measured as ether extract after acid hydrolysis by conventional Soxhlet Extraction on a SOXTHERM 2000 automatic (C. Gerhardt, Königswinter, Germany) [13].

### Short-chain fatty acids in faeces

SCFA analysis was performed as published by [17]. Briefly, faeces-water mixtures were thawed and thoroughly mixed. After centrifugation (6000 x g, 15 min), 500 μl of the supernatant were added with 50 μl *i*-caproic acid (internal standard), mixed and centrifuged (6000 x g, 15 min). For gas chromatographic measurements, 1 μl of the solution was used (Shimadzu model GC 17A, Shimadzu, Kyoto, Japan).

### Faecal water preparation

Fresh faeces samples were homogenised and diluted 1:1 with phosphate buffered saline (PBS) according to Klinder et al. [18]. Afterwards the faeces-PBS mixture was centrifuged for 4 h at 51500 x g at 4 °C. The supernatant (FW) was aliquoted and stored at − 80 °C.

### Cell culture and cytotoxicity of faecal water

In the present analysis, the human colorectal adenocarcinoma cell line HT29 (American Type Culture Collection no. HTB-38, Rockville, MD, USA) was used for in vitro experiments. The specific properties and cell culture conditions have been described previously [19]. Briefly, cells were grown in Dulbecco’s modified eagle medium (DMEM, Biochrom, Berlin, Germany), which was supplemented with 10% (*v*/v) fetal bovine serum (FBS Superior, Biochrom, Berlin, Germany) and 1% (v/v) antibiotics (penicillin/streptomycin, Biochrom, Berlin, Germany). Within about 24 h the cells doubled their number under given laboratory conditions. In the experiments, passages 10-15 were used. To exclude contamination with mycoplasma, a mycoplasma test (MycoAlert Detection Kit, Lonza, Cologne, Germany) was performed [20].

To determine the cellular vitality after FW incubation, the trypan blue exclusion test, which is based on changes in membrane integrity of the treated cells, was used [21]. For this, the harvested HT29 cell suspension was incubated with trypan blue (Trypan Blue Solution, Sigma Aldrich, Steinheim, Germany) in a 1:1 dilution, followed by automatically counting of viable and dead cells using the ViCell XR (Beckman Coulter, Brea, CA, USA).

### Genotoxicity of faecal water

Genotoxicity of FW was tested in HT29 cells as proposed by Oberreuther-Moschner et al. [22]. Briefly, cells were harvested and incubated with FW for 30 min at 37 °C. Single cell microgel electrophoresis (comet assay) was used to analyse genotoxic activity of FW. For this, cells were embedded into agarose on microscopical slides, lysed and subjected to electrophoresis [17, 23]. Each FW sample was analysed in triplicates. PBS as negative control and 75 μM H_2_O_2_ as positive control were included for each assay, in order to ensure the performance of the comet assay. Microscopic evaluation was performed using the Comet Assay IV image analysis system (Perceptive Instruments, Suffolk, UK). The extent of DNA migration was determined for 60 spots per slide. The tail intensity (TI) was used as the marker for evaluation. The TI is defined as the proportion of tail fluorescence intensity of total comet fluorescence intensity.

### DNA extraction and sequencing

A total of 30 faecal samples (five of each intervention group, thereof three men and two women) were extracted with PowerSoil® DNA Isolation Kit (MO BIO, Carlsbad, CA, USA) following the manufacturer’s protocol. DNA was quantified using a NanoDrop 2000 spectrophotometer (Thermo Scientific, Waltham, MA, USA) and stored at − 20 °C until further analysis. Illumina library preparation was performed according to Camarinha-Silva et al. [24] with a slightly modified sequence of the primer 27F (AGRGTTHGATYMTGGCTCAG). Amplicons were verified by agarose gel electrophoresis, purified and normalized using SequalPrep Normalization Kit (Invitrogen, Carlsbad, CA, USA). Samples were sequenced using 250 bp paired-end sequencing chemistry on an Illumina MiSeq platform. Raw reads were quality filtered, assembled and aligned using the Mothur pipeline [25]. A total of 36,915 ± 1233 sequencing reads were obtained per sample. The UCHIME algorithm was used to find possible chimeras, and reads were clustered at 97% identity into 832 operative taxonomic units (OTU). Only OTUs present on average abundance higher than 0.002% and a sequence length > 250 bp were considered for further analysis. The closest representative was manually identified with seqmatch from the Ribosomal Database Project classifier [26]. Sequences were submitted to the European Nucleotide Archive under the accession number PRJEB2203.

### Statistics

Data analysis was performed using the SPSS Statistics 21 statistical software package (IBM, Chicago, IL, USA). Power calculation for the primary outcome (plasma phosphate concentration) of the human intervention study was published elsewhere [10]. Variance homogeneity and normal distribution were tested using the Levene and the Kolmogorov-Smirnov test, respectively. The effect of supplementation between groups was tested using univariate analysis of variance followed by Bonferroni *post-hoc* test. Effects of time and gender were tested with paired and unpaired Students t-test, respectively. Differences were considered significant at *p* ≤ 0.05. Values in the text and tables are means with standard deviations.

The amplicon sequencing data set was standardised by total, and the community similarity structure was depicted through non-metric multidimensional scaling plots and Principal Coordinate Analysis by calculating the Bray-Curtis similarity matrix using PRIMER version 7.0.9 (PRIMER-E, Plymouth Marine Laboratory, Plymouth, UK) [27]. PERMANOVA routine, based on the pair-wise tests using a permutation method under a reduced model, was used to study the significant differences and interactions between factors (diet, time point and gender). Similarity percentages analysis (SIMPER) identified the species contribution to the Bray-Curtis similarity among samples within each diet [27]. Differences in the abundance of OTUs of interest between diets were evaluated using the unpaired Welch’s t-test that can handle unequal variances, unequal sample sizes and non-parametric data [28]. OTU abundances were considered significantly different for *p* < 0.05.

## Results

Baseline characteristics of subjects who completed the study and nutrient intake (three-day dietary record) have been published elsewhere [10]. Briefly, age, BMI, serum 25-hydroxyvitamin D, kidney function as well as intake of fat, protein and carbohydrates were not significantly different between the three study groups. Phosphorus and calcium intake increased significantly after the respective supplementations.

### Supplement tolerance questionnaire

**Fig. 2:**
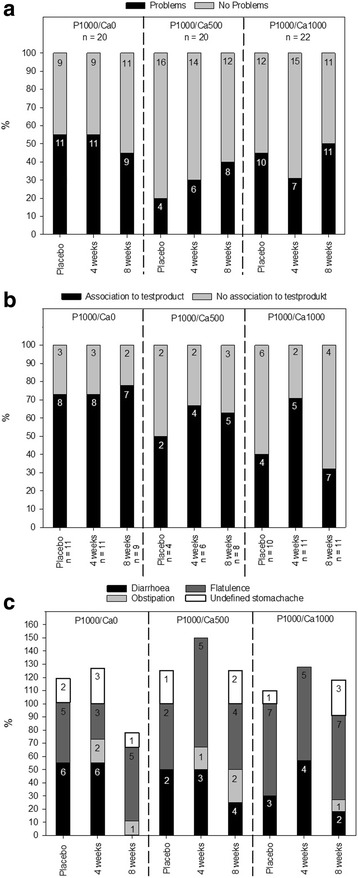
Overview of intestinal disturbances after supplementation with phosphorus and calcium. **a**: Proportional (y-axis) and absolute (numbers in the bars) distribution of subjects reporting problems with gut health or not; **b**: proportional (y-axis) and absolute (numbers in the bars) distribution of reported associations with the test product, when subjects reported complaints; **c** proportional (y-axis) and absolute (numbers in the bars) distribution of types of reported problems; P1000/Ca0: 1000 mg phosphorus/0 mg calcium; P1000/Ca500: 1000 mg phosphorus/500 mg calcium; P1000/Ca1000: 1000 mg phosphorus/1000 mg calcium

After consumption of placebo and the test products, 20-40% of subjects in each intervention group and at each time point reported complaints with gut health in the last four weeks (Fig. 2a). The majority of these subjects established a subjectively connection to the sherbet powder. This was independent of the respective phosphorus/calcium supplementation or placebo (Fig. 2b). The main complaints according to the questionnaire were diarrhoea and flatulence; a few subjects also reported obstipation and undefined stomach ache (Fig. 2c).

### Faecal pH value and fat concentration

After P1000/Ca0 and P1000/Ca500 intervention, the faecal pH value did not change (Table 1). In the whole study collective and in male subjects, the pH value decreased significantly due to P1000/Ca1000 intervention after eight weeks compared to placebo (Table 1). Noteworthy, this effect was likely caused by one male subject with a faecal pH value of 9.1 after placebo.

**Table 1:** Faecal pH values and faecal fat concentrations after intervention with phosphorus and calcium

Parameter	P1000/Ca0	P1000/Ca500	P1000/Ca1000
Faecal pH value			
n	16	18	16
Placebo	6.7 ± 0.7	6.6 ± 0.5	7.0 ± 0.7
8 weeks	6.5 ± 0.5	6.3 ± 0.5	6.5^*^ ± 0.6
Faecal fat [g/100 g fresh faeces]			
n	19	20	21
Placebo	5.9 ± 2.8	5.6 ± 3.2	4.1 ± 1.7
8 weeks	5.5 ± 3.1	5.6 ± 2.5	4.0 ± 2.3

Data are expressed as mean ± standard deviation

*P1000/Ca0* 1000 mg phosphorus/0 mg calcium, *P1000/Ca500* 1000 mg phosphorus/500 mg calcium, *P1000/Ca1000* 1000 mg phosphorus/1000 mg calcium

^*^significantly different to placebo (*p* ≤ 0.05); effect of time was tested with paired Students t-test

Faecal fat concentrations did not change significantly due to the interventions (Table 1).

### Concentration of faecal SCFA

**Fig. 3:**
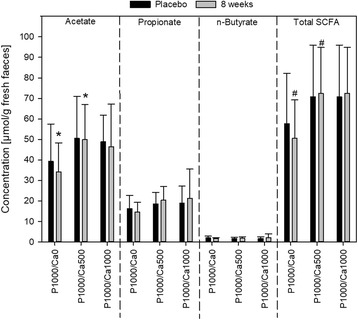
Faecal concentrations of short-chain fatty acids after supplementation with phosphorus and calcium. P1000/Ca0: *n* = 16; P1000/Ca500: *n* = 19; P1000/Ca1000: *n* = 19; data are expressed as means + standard deviations; #, * mean values with similar symbols are significantly different (*p* ≤ 0.05); effect of supplementation was tested using univariate analysis of variance followed by Bonferroni *post-hoc* test; P1000/Ca0: 1000 mg phosphorus; P1000/Ca500: 1000 mg phosphorus/500 mg calcium; P1000/Ca1000: 1000 mg phosphorus/1000 mg calcium; SCFA: short-chain fatty acids

Considering men and women together, the interventions did not affect the concentrations of total SCFA or the concentrations of the main SCFA acetate, propionate and butyrate compared to placebo (Fig. 3). Certainly, concentrations of total SCFA and acetate were significantly higher after eight weeks of P1000/Ca500 supplementation compared to the P1000/Ca0 (Fig. 3). A gender-specific analysis revealed that acetate and total SCFA concentrations were significantly higher in the P1000/Ca1000 group compared to the P1000/Ca0 group after eight weeks, but only in male subjects (Additional file 1: Figure S1). The faecal concentrations of SCFA in the female subjects did not change (Additional file 2: Figure S2).

### Cytotoxicity and genotoxicity of faecal water

**Fig. 4:**
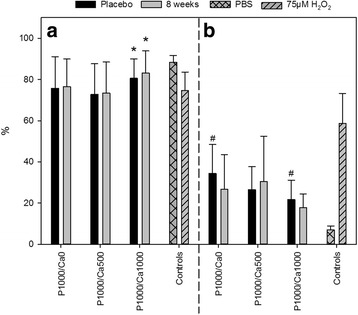
Cell vitality (**a**) and tail intensity (**b**) of HT29 cells. **a** P1000/Ca0: *n* = 15; P1000/Ca500: *n* = 19; P1000/Ca1000: *n* = 17; **b** P1000/Ca0: *n* = 10; P1000/Ca500: *n* = 8; P1000/Ca1000: *n* = 15; cell vitality and tail intensity after treatment of HT29 cells with faecal water collected following supplementation with phosphorus and calcium; data are expressed as means + standard deviations; #, * mean values with similar symbols are significantly different (*p* ≤ 0.05); effect of time was tested with paired Students t-test, effect of supplementation was tested using univariate analysis of variance followed by Bonferroni *post-hoc* test; P1000/Ca0: 1000 mg phosphorus; P1000/Ca500: 1000 mg phosphorus/500 mg calcium; P1000/Ca1000: 1000 mg phosphorus/1000 mg calcium; PBS: phosphate buffered saline

After incubation with FW, vitality of the HT29 cells ranged on average between 73 and 83%. For the P1000/Ca1000 intervention group, incubation of HT29 cells with FW from the eight-week time-point resulted in a significantly higher vitality of the cells compared to placebo (Fig. 4a). The interventions with phosphorus did not significantly affect the TI as a marker for genotoxic activity of the FW **(**Fig. 4b), considering men and women together and separately. Certainly, FW obtained from P1000/Ca0 subjects showed significantly higher genotoxic activity before the intervention compared to those from the P1000/Ca1000 group (Fig. 4b). Based on our results that PBS (negative control) caused no genotoxic damages (TI 7 ± 2%) and H_2_O_2_ caused strong genotoxic effects (TI 59 ± 15%), the FW matrix showed only moderate genotoxicity independent of the phosphorus and calcium interventions (mean TI for all subjects 25 ± 13%).

### Microbial composition in faeces

**Fig. 5:**
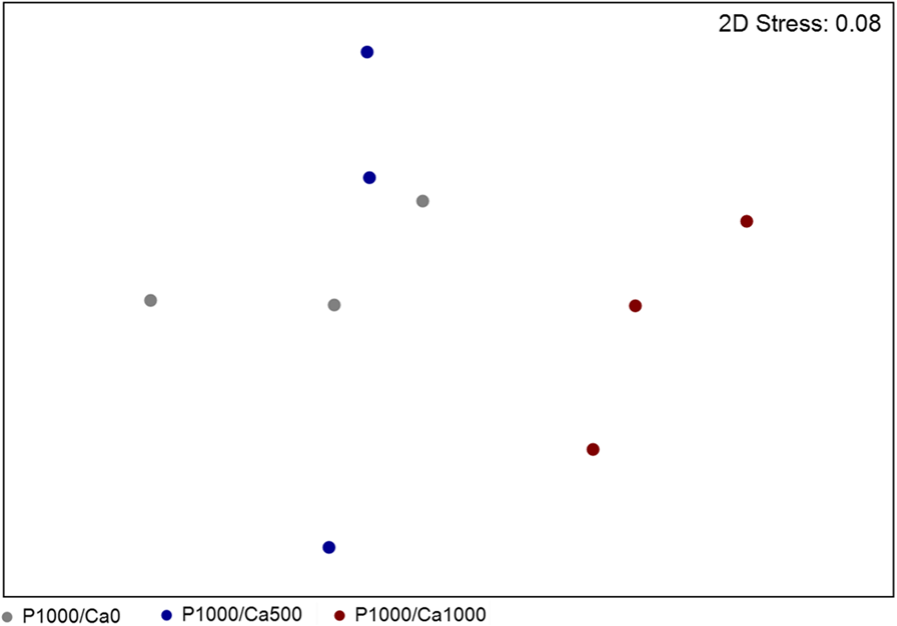
Global bacterial community structure of men after eight weeks of intervention with phosphorus and calcium. Sequencing data were standardized prior to the use of Bray-Curtis similarity algorithm, the symbols represent a unique sample comprising all OTUs and its abundance information, the data of the red symbols (P1000/Ca1000) are significantly different from the grey (P1000/Ca0, *R* = 0.778, *p* = 0.01) and blue (P1000/Ca500, *R* = 0.593, *p* = 0.01) ones, P1000/Ca0: 1000 mg phosphorus; P1000/Ca500: 1000 mg phosphorus/500 mg calcium; P1000/Ca1000: 1000 mg phosphorus/1000 mg calcium, *n* = 9; ns: not significant, OTU: operational taxonomic unit

**Fig. 6:**
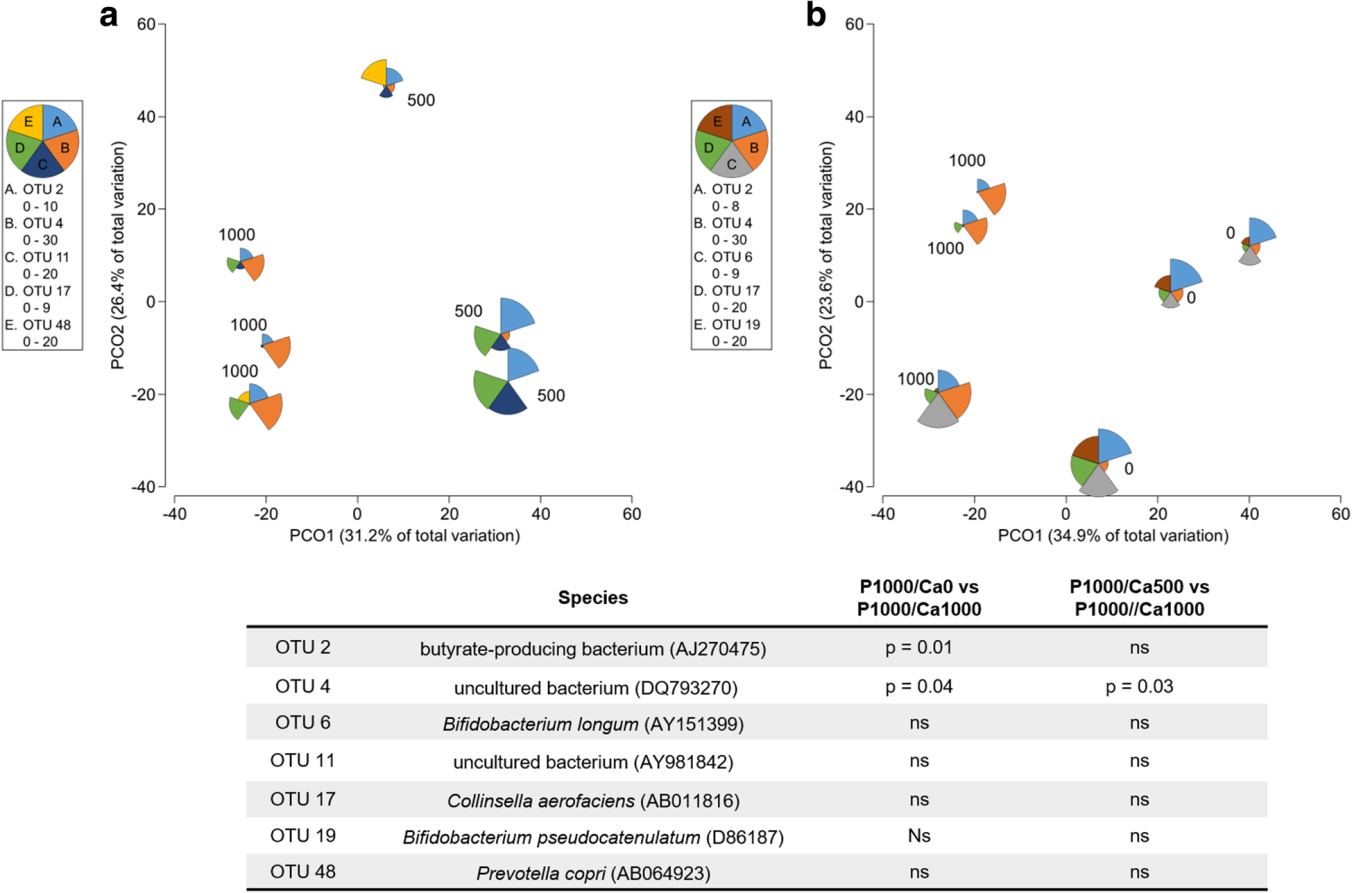
Principal coordinate analysis ordination of the global microbial community structure of men**. a** Data of men after eight weeks of P1000/Ca1000 vs. P1000/Ca500 intervention; **b** data of men after eight weeks of P1000/Ca1000 vs. P1000/Ca0 intervention, *n* = 6; bubbles were superimposed to visualise the relative abundance of the most relevant OTUs; P1000/Ca0: 1000 mg phosphorus; P1000/Ca500: 1000 mg phosphorus/500 mg calcium; P1000/Ca1000: 1000 mg phosphorus/1000 mg calcium, ns: not significant, OTU: operational taxonomic unit; PCO: principal coordinate

Considering all subjects of the study subgroups (*n* = 5 for each intervention), no differences of the microbiome were observed between placebo and eight weeks of intervention as well as between the different intervention groups after eight weeks. Interestingly, men of the P1000/Ca1000 intervention had a significantly different gut microbial community compared to the men of the P1000/Ca0 (*R* = 0.778, *p* = 0.01) and the P1000/Ca500 groups (*R* = 0.593, *p* = 0.01) (Fig. 5). Abundance of the genus *Acetivibio* and of the family *Coriobacteriaceae* were significantly lower in men after eight weeks of P1000/Ca500 intervention when compared to P1000/Ca0 intervention (*p* < 0.05). *Clostridium* XVIII was significantly more abundant in men of the P1000/Ca1000 intervention group compared to men of the P1000/Ca500 (*p* = 0.04) and P1000/Ca0 (*p* = 0.04) groups. The OTUs belonging to *Bifidobacterium* genus were significantly less abundant in faeces of men of the P1000/Ca1000 intervention compared to the faeces of men of the P1000/Ca500 group (*p* = 0.04). Figure 6 shows the relative abundance of the OTUs contributing to the group separation of the men per intervention after eight weeks. OTU4, an uncultured bacterium related to *Clostridium* XVIII, was significantly more abundant in the men of P1000/Ca1000 intervention group compared to P1000/Ca500 (*p* = 0.03) and P1000/Ca0 (*p* = 0.04). OTU2, a butyrate-producing bacterium, was significantly more abundant in men of the P1000/Ca0 intervention group compared to the men with P1000/Ca1000 supplementation (*p* = 0.01). A trend was observed when the microbial community of men was compared with the one of women (P1000/Ca1000) (*p* = 0.1). OTU 4 was more abundant in men while OTU 19 and OTU 28 were more abundant in women (*p* < 0.03) (Additional file 3: Figure S3). Dietary interventions in women did not cause any significant differences of the microbial community.

## Discussion

Already in the 1980s, Newmark et al. hypothesized that calcium ions, bile and fatty acids form insoluble soaps in the human gut, which are detectable in the faeces [29]. Initially, phosphate was considered as a competitor of bile and fatty acids, but Van der Meer et al. showed that phosphate did not compete with bile and fatty acids, but was rather necessary for the precipitation with calcium [30]. At a molar calcium-to-phosphate ratio of 3:2 and a pH value of 5.6, one part of the ingested dietary calcium and phosphate precipitates as ACP in the small intestine [31]. ACP are able to precipitate both glycine-conjugated and free bile acids by ionic absorption of their negatively charged carboxyl group to the positively charged calcium ions on the surface of the ACP [31]. The hydrophobic tail of the bile acid is exposed to the aqueous phase, which in turn leads to the formation of hydrophobic aggregates. These aggregates are able to bind other hydrophobic ligands [31]. This and the facts that calcium from milk and fermented milk products increases the resistance of rats to salmonella infections [32] and stimulates intestinal lactobacilli colonisation [33], leads to the assumption that dietary calcium and phosphorus can beneficially modify the intestinal environment.

Our human intervention study showed that the faecal concentrations of phosphorus and calcium increased significantly after eight weeks of phosphorus and calcium supplementation [10]. This increase could be due to the formation of ACP. However, even in the group supplemented only with phosphorus indications for ACP formation were observed. We found increasing concentrations of faecal phosphorus without increasing calcium concentrations, but decreasing renal calcium excretion [10]. These results led to the assumption that dietary calcium partly precipitates with phosphate to ACP and cannot be absorbed in the gut. In order to maintain calcium homeostasis of the body, fewer calcium was excreted via urine.

Formation of ACP is a well-known process in the human gut [12, 13, 28], and could be a prerequisite for a beneficial modulation of gut health. A decrease of the available intestinal bile acids by precipitation with ACP can lead to a modified microbiota composition and accordingly to a change in SCFA production and composition [34, 35]. The faecal SCFA concentrations of our subjects showed no effect over time (placebo vs. eight weeks of each intervention). But, after eight weeks, the P1000/Ca0 group showed the lowest concentrations of total SCFA (55 ± 18 μmol/g faeces) compared to P1000/Ca500 (76 ± 22 μmol/g faeces, *p* ≤ 0.05) and P1000/Ca1000 (73 ± 31 μmol/g faeces, *p* = 0.081). This higher concentration of total SCFA in the calcium supplemented groups is likely due to a higher acetate concentration. Furthermore, men showed significantly higher total SCFA and acetate concentrations after eight weeks of P1000/Ca1000 intervention compared to the P1000/Ca0 one. These effects are independent of the water content of the faeces, since faecal dry matter did not change due to the interventions (data not shown). In a study of Ditscheid, total SCFA (*p* = 0.065) and acetate concentrations (*p* = 0.019) increased after supplementation with calcium phosphate (β-tricalciumphosphate) compared to placebo [36]. The author assumed that either an increased production or a decreased absorption of acetate could be the reason for the higher faecal concentrations after calcium phosphate supplementation**.** Trinidad et al. showed that bioavailable calcium supports intestinal acetate absorption [37]. Therefore, Ditscheid hypothesized that bioavailability of calcium decreased due to ACP formation and thus acetate absorption is diminished [36]. But, calcium phosphate supplementation has been shown to modulate the intestinal gut community by changing the intestinal environment [11, 33]. This shift of the gut microbiota could lead to an increased production of SCFA, such as acetate [34].

In the present study, we determined in a subgroup of five subjects per intervention the microbial community in faeces by DNA extraction and amplicon sequencing. Interestingly, men of the P1000/Ca1000 group showed a shift of the microbial community compared to men of the two other intervention groups. *Clostridium* XVIII was significantly more abundant in men of the highest calcium group. Furthermore, men of the P1000/Ca1000 intervention group had significantly higher acetate concentrations compared to men of the P1000/Ca0 one. In general, *Clostridium spp*. are known to produce acetate by microbial fermentation of carbohydrates in the human gut [34]. Although, the detected *Clostridium* XVIII is an uncultured bacterium and not characterised so far, it could exhibit similar acetate producing properties. Moreover, OTU2, a butyrate-producing bacterium, was significantly more abundant in men of the P1000/Ca0 intervention group and tended to be more abundant in the P1000/Ca500 group compared to the men of the P1000/Ca1000 (*p* = 0.01) one. But whether these associations depend on the calcium and phosphorus supplementation remains unclear, since we were only able to study the microbial community of a subgroup of five subjects per intervention. The partly gender-specific effects might be a result of sex-specific diet effects on the human microbiome as proposed by Bolnick et al. [38]. Animal and human studies showed, that gender influences microbial composition in the gut [38–41] and, thus, the formation of SCFA [42], too.

A modulation of the cyto- and genotoxicity of the FW after calcium and phosphorus supplementation is discussed since ACP are known to precipitate secondary bile acids in the gut [29, 43]. Secondary bile acids showed cyto- and genotoxic effects on human normal and tumour colon cells [44–46]. Our results indicate that the phosphorus supplementation with and without calcium did not affect the genotoxicity of FW and did not markedly effect the vitality of HT29 cells. Similar results were provided by Ditscheid et al., which could not show changes in the genotoxicity of FW after calcium phosphate supplementation [47]. Glinghammer et al. reported that cytotoxicity of FW increased after subjects switched from a dairy product-rich (high calcium) to a dairy product-free diet (lower calcium), but genotoxicity did not alter [48]. The authors suggested that the mechanism by which dairy products may act colon cancer protective are at the level of tumour promotion and indicated that dairy products, especially calcium and phosphate, exert protective effects by precipitating bile and fatty acids [48]. Gomes et al. summarised in a review that high calcium diets reduce FW cytotoxicity by precipitating cytotoxic surfactants, which results in a lower colonic epithelium damage and higher resistance against infections [49]. All studies cited in this context used a calcium phosphate supplement, but almost all are based on animal models. Furthermore, the cell model systems and methods used were different to the ones used here, e.g. cytotoxicity was tested with erythrocytes [43] instead of intestinal cells. Besides, maybe a more realistic picture of the FW genotoxicity would be displayed by a quantitative stool sampling over one or more days. Unfortunately, parameters in faeces were not the main outcome of our human intervention study and so only one stool sample was collected.

Like the precipitation of bile acids, ACP are in principle able to precipitate fatty acids as well [49]. The resulting increased faecal excretion of fat could favour weight control [50]. Our study did not show changes in faecal fat concentrations in response to the phosphorus and calcium supplementation. Ditscheid et al. also reported no significant changes in faecal fat excretion after supplementation with β-tricalciumphosphate compared to a normal calcium intake (approx. 2200 vs. 1200 mg calcium/d and 2000 vs. 1600 mg phosphorus/d) [13]. In contrast, Boon et al. showed a non-significant 57% increase in faecal fat after high compared to low calcium intakes (2500 vs 400 mg calcium/d, low-fat dairy) [51]. Furthermore, in a study by Bendsen et al. faecal fat increased from 5.4 to 11.5 g/d after intake of 1600 mg calcium/d (by dairy supplementation) [52]. Another study comparing low (approx. 500 mg calcium/d) with high calcium diets (approx. 1800 mg calcium/d, low-fat dairy products) showed a 2.5-fold increase in faecal fat after the high calcium intake [53]. One reason for the discrepancies could be the standardising of the diet and stool collection time. In the study presented here a dietary record for three days was done and one faecal sample was collected. However, the study of Ditscheid et al. included a standardised diet and a five-day faecal collection. But, nevertheless they also found no effect on faecal fat concentration. Possibly, this could be caused by the normal calcium intake that was used as placebo here (approx. 900 mg calcium/d) [10] and by Ditscheid et al. (approx. 1200 mg calcium/d) [13], whereas in the studies by Boon et al. (400 mg calcium/d) [51] and Jacobsen et al. (500 mg calcium/d) [53] low calcium diets were used as controls. It is therefore possible that the observed changes in faecal fat concentrations were caused by decreasing the intake of calcium in the controls to levels below the norm.

In a phosphate supplementation study of Grimm et al., a phosphate-rich diet (additional 1436 mg phosphorus/d) was associated with intestinal distress, soft stool or mild diarrhoea. The authors concluded that the symptoms are a consequence of an osmotic effect of the added polyphosphates in the intestinal lumen [15]. In our study 20-40% of the subjects in each intervention group and at each time point reported intestinal disturbances. The majority of the subjects mentioned a connection to the test product. Analysis of the questionnaires showed that the reported disturbances were independent of the phosphorus and calcium supplementation and time-point of intervention. Therefore, we consider the gut problems as a result of the sherbet powder consumption in general and not as a result of the phosphorus and calcium supplementation. Another reason could be the focus of the subjects on the new diet component (sherbet powder) and a resulting overestimation of intestinal distress in case of gut problems. Furthermore, the interpretation of the questionnaires is limited, since the subjects reported only whether they had problems but not the frequency and intensity of disturbances.

The most important limitations of our study are (i) the restricted one portion faecal collection and (ii) the determination of gut community in a limited subgroup with only a few subjects.

To the best of our knowledge, this is the first human intervention study in the field of high phosphorus and calcium intakes, which also paid attention on gut health. As the main part of the additional phosphorus and calcium passes the whole gut via ACP and seems to modulate gut physiology, further human intervention studies in this context are needed.

## Conclusions

In conclusion, the high phosphorus supplementation did not influence cyto- and genotoxicity of FW as well as the concentrations of faecal fat independent of calcium intake. Our study provides first hints for a potential phosphorus-induced modulation of the gut community and the faecal total SCFA content. Since additional dietary phosphorus and calcium passes the gut as ACP, further studies are needed to evaluate the effect of high phosphorus and calcium diets on gut-related parameters.

## Additional files

Additional file 1: Figure S1. Faecal concentrations of short-chain fatty acids after supplementation with phosphorus and calcium of men. P1000/Ca0: *n* = 7; P1000/Ca500: *n* = 9; P1000/Ca1000: *n* = 8; data are expressed as means + standard deviations; #,* mean values with similar symbols are significant different (*p* ≤ 0.05); effect of supplementation was tested using univariate analysis of variance followed by Bonferroni *post-hoc* test; a, b significantly different (Wilcoxon sign-rank test); P1000/Ca0: 1000 mg phosphorus; P1000/Ca500: 1000 mg phosphorus/500 mg calcium; P1000/Ca1000: 1000 mg phosphorus/1000 mg calcium; SCFA: short-chain fatty acids. (PNG 36 kb)

Additional file 2: Figure S2. Faecal concentrations of short-chain fatty acids after supplementation with phosphorus and calcium of women. P1000/Ca0: *n* = 9; P1000/Ca500: *n* = 10; P1000/Ca1000: *n* = 11; data are expressed as means + standard deviations; #,* mean values with similar symbols are significant different (*p* ≤ 0.05); effect of time was tested with paired Students t-test; effect of supplementation was tested using univariate analysis of variance followed by Bonferroni *post-hoc* test; P1000/Ca0: 1000 mg phosphorus; P1000/Ca500: 1000 mg phosphorus/500 mg calcium; P1000/Ca1000: 1000 mg phosphorus/1000 mg calcium; SCFA: short-chain fatty acids. (PNG 35 kb)

Additional file 3: Figure S3. Principal coordinate analysis ordination of the global community structure of men and women after eight weeks of P1000/Ca1000 intervention. *n* = 5, bubbles were superimposed to visualise the relative abundance of the most relevant OTUs; P1000/Ca1000: 1000 mg phosphorus/1000 mg calcium, ns: not significant, OTU: operational taxonomic unit; PCO: principal coordinate. (TIFF 576 kb)

## Abbreviations

ACP: Amorphous calcium phosphate
FW: Faecal water
OTU: Operational taxonomic unit
P1000/Ca0: 1000 mg phosphorus
P1000/Ca1000: 1000 mg phosphorus/1000 mg calcium
P1000/Ca500: 1000 mg phosphorus/500 mg calcium
PBS: Phosphate buffered saline
PCO: Principal coordinate
SCFA: Short-chain fatty acids
TI: Tail intensity
