# Supplementary figures and images for: High phosphorus intake and gut-related parameters – results of a randomized placebo-controlled human intervention study
Source: Nutr J. 2018 Feb 16;17:23. doi: 10.1186/s12937-018-0331-4 (PMC5815223; doi:10.1186/s12937-018-0331-4)

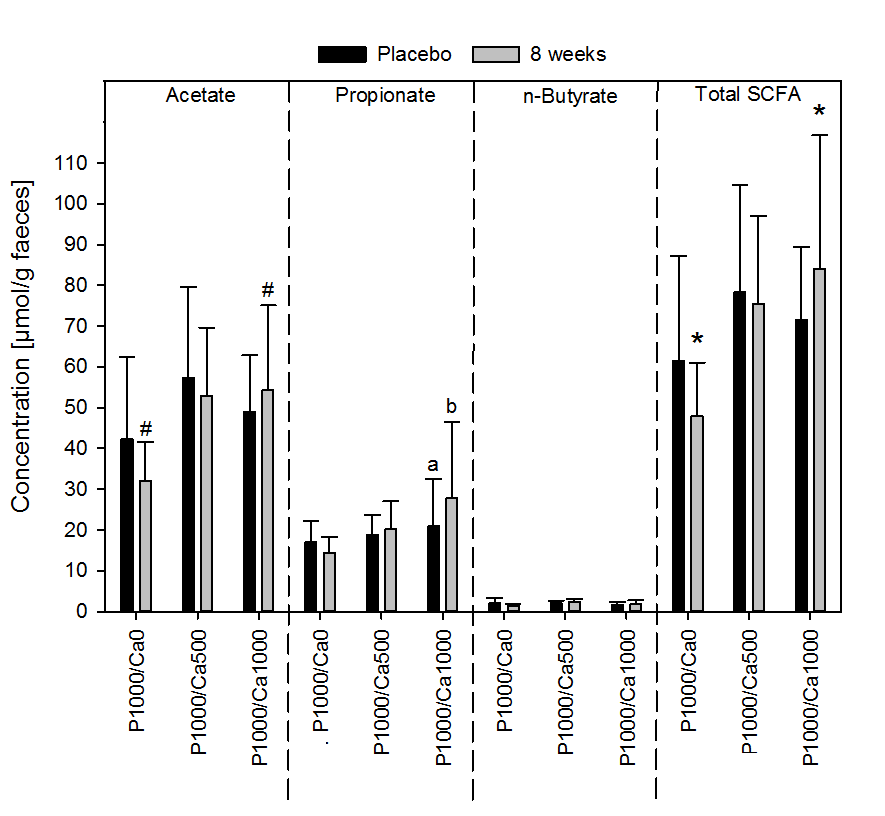

Supplement: Supplementary file 1 — Faecal concentrations of short-chain fatty acids after supplementation with phosphorus and calcium of men. P1000/Ca0: n = 7; P1000/Ca500: n = 9; P1000/Ca1000: n = 8; data are expressed as means + standard deviations; #,* mean values with similar symbols are significant different (p ≤ 0.05); effect of supplementation was tested using univariate analysis of variance followed by Bonferroni post-hoc test; a, b significantly different (Wilcoxon sign-rank test); P1000/Ca0: 1000 mg phosphorus; P1000/Ca500: 1000 mg phosphorus/500 mg calcium; P1000/Ca1000: 1000 mg phosphorus/1000 mg calcium; SCFA: short-chain fatty acids. (PNG 36 kb) [file 12937_2018_331_MOESM1_ESM.png]

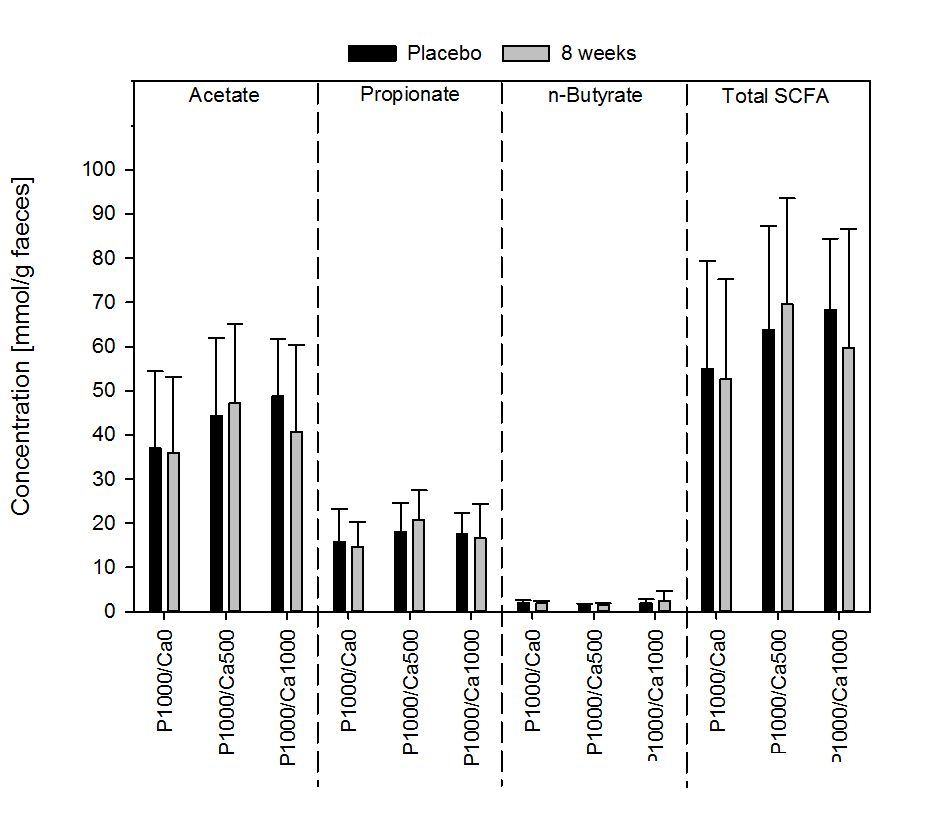

Supplement: Supplementary file 2 — Faecal concentrations of short-chain fatty acids after supplementation with phosphorus and calcium of women. P1000/Ca0: n = 9; P1000/Ca500: n = 10; P1000/Ca1000: n = 11; data are expressed as means + standard deviations; #,* mean values with similar symbols are significant different (p ≤ 0.05); effect of time was tested with paired Students t-test; effect of supplementation was tested using univariate analysis of variance followed by Bonferroni post-hoc test; P1000/Ca0: 1000 mg phosphorus; P1000/Ca500: 1000 mg phosphorus/500 mg calcium; P1000/Ca1000: 1000 mg phosphorus/1000 mg calcium; SCFA: short-chain fatty acids. (PNG 35 kb) [file 12937_2018_331_MOESM2_ESM.png]

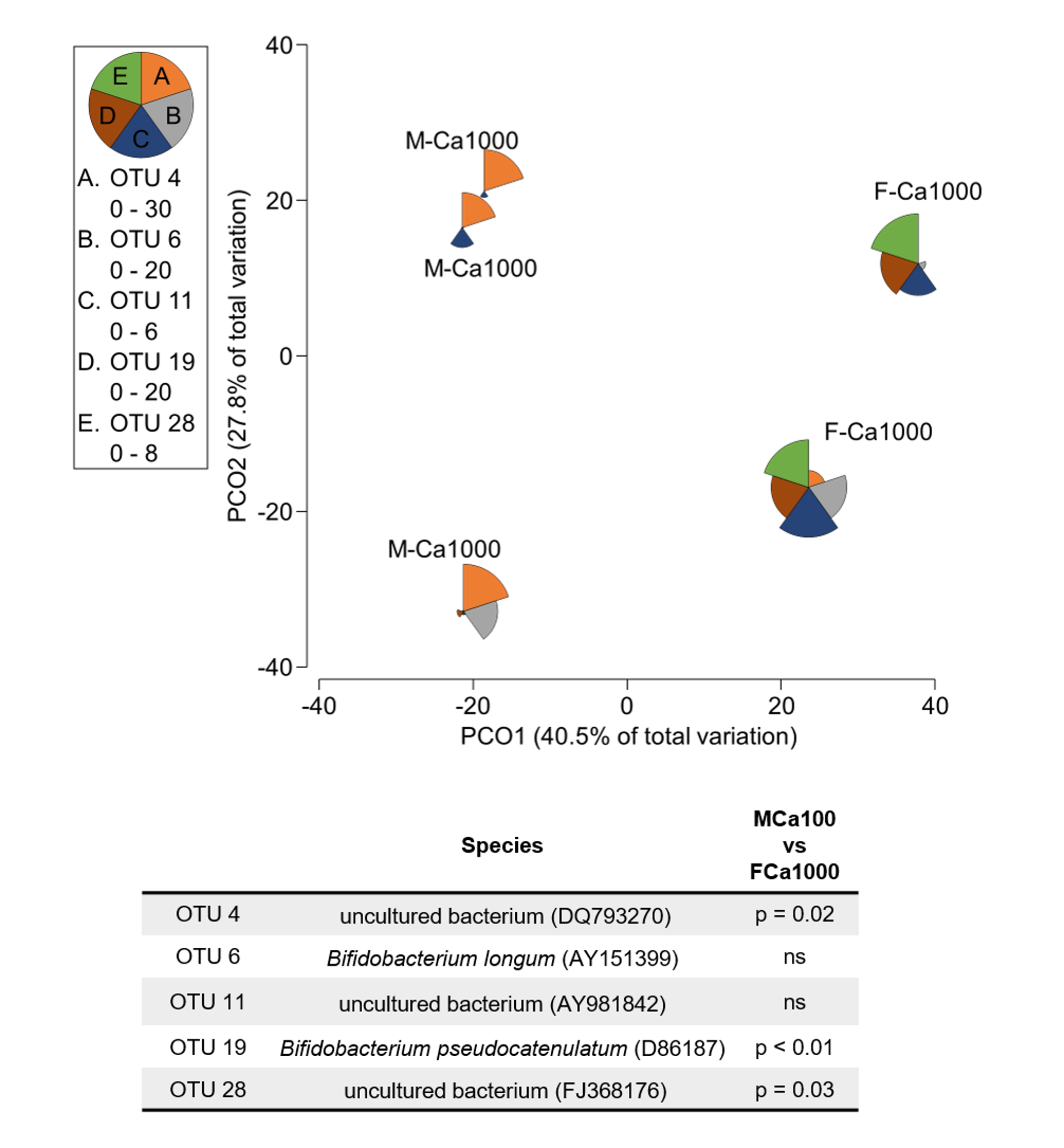

Supplement: Supplementary file 3 — Principal coordinate analysis ordination of the global community structure of men and women after eight weeks of P1000/Ca1000 intervention. n = 5, bubbles were superimposed to visualise the relative abundance of the most relevant OTUs; P1000/Ca1000: 1000 mg phosphorus/1000 mg calcium, ns: not significant, OTU: operational taxonomic unit; PCO: principal coordinate. (TIFF 576 kb) [file 12937_2018_331_MOESM3_ESM.tif]
